# Finding the place for nutrition in healthcare education and practice

**DOI:** 10.1136/bmjnph-2023-000692

**Published:** 2024-05-23

**Authors:** Ebiambu Agwara, Kathy Martyn, Elaine Macaninch, Wanja Nyaga, Luke Buckner, Breanna Lepre, Celia Laur, Sumantra Ray

**Affiliations:** 1 NNEdPro Global Institute for Food, Nutrition and Health, Cambridge, UK; 2 School of Biomedical Sciences, Ulster University, Coleraine, UK; 3 Fitzwilliam College, University of Cambridge, Cambridge, UK

**Keywords:** nutrition assessment

## Abstract

**Background:**

Malnutrition continues to impact healthcare outcomes, quality of life and costs to healthcare systems. The implementation of nutrition care in healthcare practice may improve health outcomes for patients and the community. This paper describes the iterative development and implementation of nutrition medical education resources for doctors and healthcare professionals in England. These resources are part of the Nutrition Education Policy for Healthcare Practice initiative.

**Method:**

Action research methodology was employed to develop and implement nutrition education workshops for medical students and doctors. The workshop was developed iteratively by an interdisciplinary project team, and the content was initially based on the General Medical Council outcomes for graduates. It was evaluated using quantitative evaluation tools and informal qualitative feedback captured from attendees using tools provided by the host organisations and developed by the roadshow team.

**Results:**

A total of 6 nutrition education workshops were delivered to 169 participants. This simple educational package demonstrated potential for delivery in different healthcare settings; however, formal feedback was difficult to obtain. Evaluation results indicate that workshops were better received when delivered by doctors known to the participants and included local context and examples. Reported barriers to the workshops included difficulty for participants in finding the time to attend, beliefs that peers gave a low priority to nutrition and uncertainty about professional roles in the delivery of nutrition care.

**Conclusion:**

A key outcome of this project was the development of resources for nutrition training of doctors, adapted to local needs. However, relatively low attendance and multiple barriers faced in the delivery of these workshops highlight that there is no ideal ‘place’ for nutrition training in current healthcare teaching. Interprofessional education, through relevant clinical scenarios may increase awareness of the importance of nutrition in healthcare, support the alignment of health professional roles and improve subsequent knowledge and skills.

**WHAT IS ALREADY KNOWN ON THIS TOPIC**

- There is a great need for more nutrition within medical education, as well as a need for greater clarity of a doctor’s role in nutritional care and when to refer for specialist advice.

**WHAT THIS STUDY ADDS**

- The development and implementation of resources for nutrition training of doctors adapted to local needs.
- This paper shows that there is no one ‘place’ for nutrition; hence, the Nutrition Implementation Coalition provides a ‘hub’ of material and expertise adapted to the needs of the providers and settings.

**HOW THIS STUDY MIGHT AFFECT RESEARCH, PRACTICE OR POLICY**

- The need for multiprofessional ‘hub’ of material and expertise that can support medical schools and healthcare professionals who may lack faculty to develop and implement nutrition education in practice.

## Introduction

Malnutrition comprises the double burden of undernutrition and overnutrition including micronutrient deficiencies, which together continue to impact the healthcare outcomes and quality of life of individuals, as well as costs to the healthcare systems.^1^ For example, in hospitals, rates of malnutrition remain high, averaging 35% internationally, and in 2015, it was estimated that malnutrition in England cost the National Health Service (NHS) almost £20 billion.^2^

Implementing nutritional care in practice requires the application of nutrition knowledge and skills, and ideally this care is individualised to health priorities, patients’ goals, preferences and sociocultural context.^3^ Dietitians are specifically trained to provide nutrition care; however, due to their limited numbers, they rely on nutritional problems being recognised by others with subsequent clinical referral. Furthermore, other health professionals, such as doctors and nurses, are well placed to initiate nutrition care and provide support of advice as they tend to have regular contact with patients and doctors are perceived by patients as a credible source of nutrition information.^4^ This provides opportunities for discussions and nutrition screening.^5^

However, although doctors, nurses and other health professionals perceive nutrition as important, they require the knowledge, skills and confidence to incorporate nutrition as part of patient care or to identify when an individual might benefit from a referral to a dietitian. Importantly, medical students and doctors' welcome further nutrition education, as professional bodies internationally now recommending doctors discuss diet with their patients.^6–8^ Despite this perceived need, and continual focus on improving medical nutrition education, undergraduate or preregistration nutrition education for doctors is limited.^9^ Moreover, there is limited information available on nutrition learning objectives or outcomes, or teaching methods. Internationally, only 45% of medical education accreditation and curriculum guidance was found to even mention nutrition,^10^ and to this end, there is limited incentive for education providers to include nutrition in medical training.

In the UK, the responsibility for postgraduate medical education (PGME) is devolved to the respective professional bodies in England, Wales, Scotland and Northern Ireland.^11^ The programmes are commonly referred to as foundation programmes, core training and specialty training.^12^ During foundation training, junior doctors have protected learning time, and nutrition is identified as part of the syllabus followed in England, Wales, Scotland and Northern Ireland.^13^ Separate curriculum exists for 32 specialty training programmes in the UK.^14^ In specialty PGME, nutrition content varies depending on the perceived relevance of nutrition to the medical specialty with limited mandated content. Table 1 shows examples of where nutrition is mandated in UK postgraduate medical curriculum. In 2021, with the aim to standardise nutrition education in undergraduate medical training, a working party convened by the Association for Nutrition (AfN) published the nutrition curriculum for UK undergraduate medical students.^15^ This builds on the UK General Medical Council (GMC) ‘outcomes for graduates’,^16^ which stipulates the core competencies for medical graduates in the UK. However, ways to support systematic integration of nutrition into medical education are required, as there is currently no requirement to include nutrition in medical training. Even if nutrition education can be integrated into current medical training, there remains a need for nutrition education and ongoing support for medical doctors who are already qualified.^17^

**Table 1:** Examples of where nutrition is mandated in UK postgraduate medical curriculum

The UK Foundation Programme Curriculum 2021^13^	The recognition and assessment of eating disorders. Appropriately instigates nutritional assessments. The need to embed health promotion within practice to improve population health is highlighted in reference to social prescribing.
The RCGP Curriculum The Curriculum Topic Guides 2020^20^	Gastroenterology: the role of nutrition in both the prevention and treatment of gastrointestinal disorders, including the correction of nutritional problems. Metabolic problems and endocrinology: confidence in providing health promotion advice. Also, in giving specific nutritional advice alongside prescribing and altering of medication within diabetes and obesity treatment. Population health: promoting health and preventing disease. Awareness of local and national guidelines on obesity and malnutrition and the relevance of nutrition in national screening programmes such as the National Health Service health check and older adult screening. Smoking, alcohol and substance misuse: recognising and treating malnutrition and vitamin deficiencies as a medical complication of substance misuse and within treatment for associated neurological complications. People at the end of life: ethics around food and hydration within end of life care. Allergy and immunology: recording of food sensitivities. Recognising food allergies and appropriate diagnosis, the economic and psychosocial effect on the individual and caregivers/social network.
Geriatric Medicine 2022 Curriculum^22^	**Nutrition**: to know how to assess the nutritional status of older people in different care settings and in conjunction with other relevant health professionals be able to devise an appropriate nutritional support strategy for patients. Specific reference to nutrition is made in stroke, dementia and palliative care.

*Nutrition content identified by searching the above curriculum documents for the keywords nutrition, diet or food.

With these challenges in mind, in March 2019, the NNEdPro Global Institute for Food, Nutrition and Health, which has a key focus on medical nutrition education, launched a nutrition education package as part of their ‘Nutrition Education Policy in Healthcare Practice (NEPHELP)’ project. The aims of NEPHELP were: (1) to develop, evaluate and implement nutrition education workshops and educational resources; (2) to understand the feasibility and acceptability of a nutrition education model via participant and facilitator feedback; and (3) to gain insights into where doctors and health professionals see the place for nutrition in their education. This paper primarily focuses on aim 1, and to a lesser extent, secondarily focuses on aims 2 and 3.

This paper describes the iterative development and delivery of a nutrition education workshop for junior doctors and health professionals piloted in Glasgow, then delivered at six sites across England. Feasibility and acceptability of the workshops are explored along with reflections on the place for nutrition in medical and healthcare profession education.

## Methodological approach

### Study design

The development of NEPHELP used action research methodology,^18^ which is considered a pragmatic approach to instigate change. The action research cycle includes problem identification (including reflection), planning, action (implementation of change and monitoring) and evaluation or reflection before starting a new situation analysis. Action research was considered rigorous in this context because it supported the aim of exploring both enablers and barriers to the implementation of nutrition education in medical practice with the research participants.^19^

**Figure 1:**
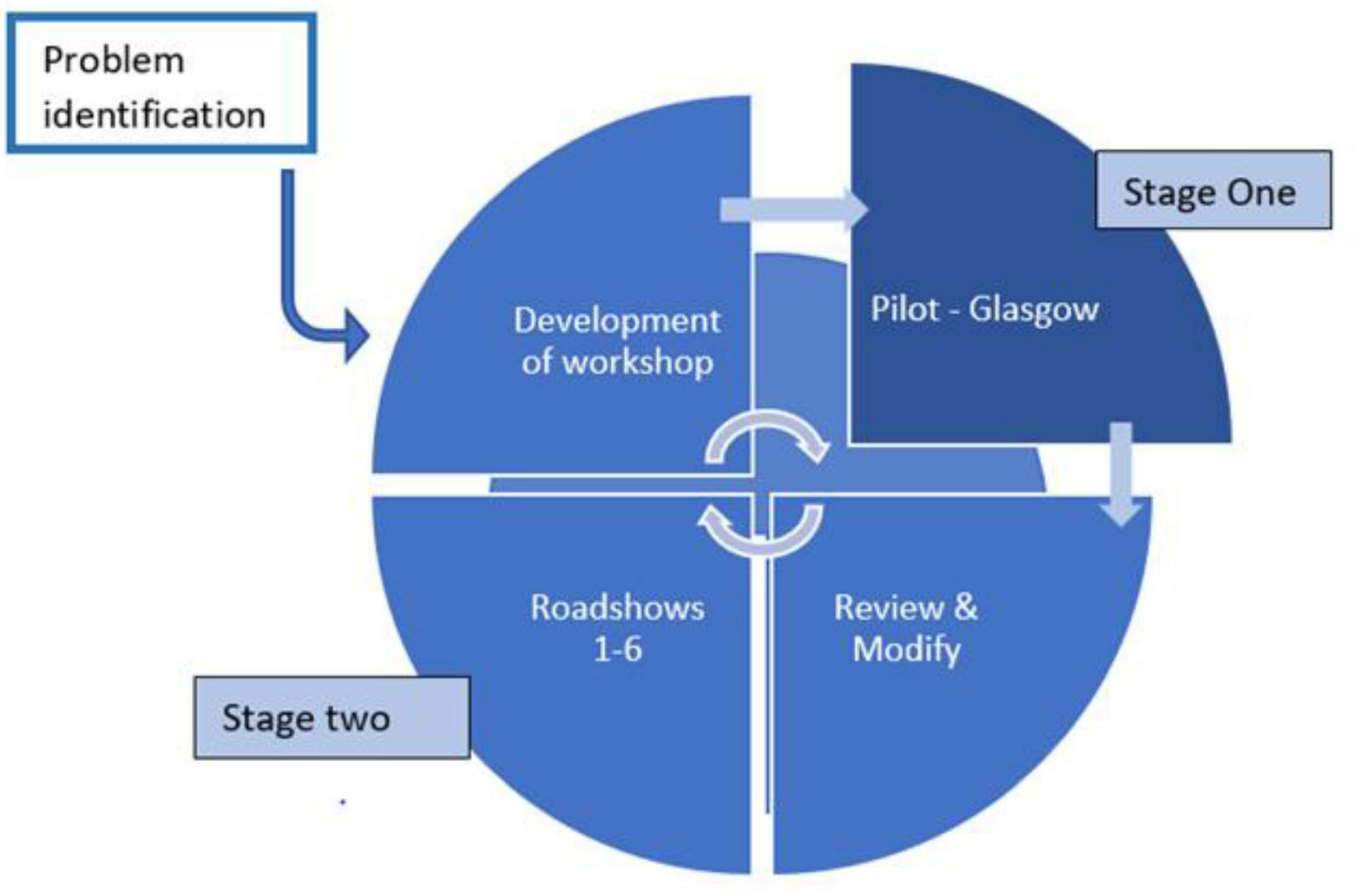
Place for nutrition paper.

The project was conducted in two stages, including (1) an initial pilot workshop in Glasgow, followed by (2) the delivery of workshops across England as part of the ‘NEPHELP Nutrition Training Roadshow’ (figure 1).

### The NEPHELP team

The interdisciplinary teaching team consisted of medical doctors, a registered dietitian, associate and registered nutritionists, a registered nurse, academics and education professionals, all members of the NNEdPro Global Institute. The teaching team developed and delivered the workshops and the evaluation tool.

### Stage 1: piloting the NEPHELP workshop

#### Workshop participants and recruitment

This was conducted with a multiprofessional international audience attending the *BMJ Quality and Safety* in Healthcare conference at Glasgow in 2019. Participants voluntarily signed up for a 4-hour NEPHELP nutrition workshop.

#### Workshop content

The initial workshop content was based on GMC expected learning outcomes for medical graduates,^16^ postgraduate curricula^13 20^ and perceived nutrition priorities within clinical settings, based on the educational and professional experiences of the interdisciplinary team, and included an overview of nutrition science related to clinical care Case-based discussions were used to assist with the translation of nutrition knowledge into practice.

The workshop aimed for participants to: (1) understand key principles of human nutrition, (2) understand the importance of balanced diet for health, and in illness, recognise factors that impact on equitable access to healthy food, (3) explore how individuals can include nutritional screening, care and dietetic referral into their practice and (4) provide feedback on the acceptability of the workshop as a strategy for delivering nutrition education.

#### Evaluation

The NEPHELP evaluation tool included quantitative and open-ended questions, which were designed to explore the knowledge, attitude and practices in nutritional care, and perceptions of requirements for nutrition in medical education. Feedback on the workshop content and ideas on how the programme could be improved were also gathered to inform further development of the workshop.

### Stage 2: delivering the NEPHELP ‘Nutrition Training Roadshow’ across England

In this second stage, termed the ‘roadshow’, the NEPHELP workshop was delivered, evaluated and adapted to six healthcare settings across England. The NEPHELP roadshow was delivered in three formats: (1) as a part of mandatory training within health settings (hospitals, primary care etc), (2) a standalone workshop or (3) as an invited workshop delivered as part of a general GP training day and organised by an external group.

#### Participants and recruitment

Workshop participants were invited by the PGME and NHS contacts who also circulated workshop information. The delivery of stage 2 workshops varied depending on NEPHELP facilitator availability, with some being codelivered between the NEPHELP team and local dietitians or doctors.

#### Evaluation

Following each iteration of the workshop, evaluation forms and informal reflections were captured to inform the refinement of workshop content. The NEPHELP evaluation tool (described above) was adapted to the audience in each workshop. Additional feedback was provided by PGME faculty through their own evaluation forms. This ‘in-house’ feedback differed for each centre, was designed and collected independently but shared with the NEPHELP team. NEPHELP observers took notes during workshop discussions, and after each workshop, the facilitators listened to the observations and critically reflected on the session.

### Analysis

The iterative nature of the action research methodology allowed for the continuous review of data as it was collected. Quantitative data collected through NEPHELP evaluation tool and PGME forms were analysed descriptively. Qualitative data collected from open questions in the evaluation tool, PGME forms, notes taken during the workshop by observers and postworkshop critical reflection sessions were subject to content analysis,^21^ an approach widely used in qualitative research. Analysis was completed by two members of the NEPHELP team, who did not have prior relationships with any of the participants. EM and KM independently grouped the material into topics using a word processing package, and the main topics were then discussed with the wider NEPHELP team.

## Results

The initial 4-hour workshop was piloted in Glasgow in March 2019, with 40 interdisciplinary participants at the *BMJ Quality and Safety* in Healthcare conference. Following the pilot, the workshop was modified based on participant evaluations (n=4), oral feedback from participants and the NEPHELP team reflections. The roadshow was conducted between March 2019 and February 2020, with workshops conducted at 6 locations across England with 169 participants and a total of 13.5 hours (table 2).

**Table 2:** Overview of completed NEPHELP workshops (March 2019 to February 2020)

Workshop location	Audience	Setting	Voluntary/mandated	Number attending	Duration
*BMJ Health and Safety* conference (pilot)	International multidisciplinary	Multiple	Voluntary	40	4 hours
South East England	FY2 doctors in secondary care	Hospital	Mandated	33	2 hours
South East England	General practitioner trainees	Primary care	Mandated	40	2 hours
Essex	Senior general practitioner	Primary care	Voluntary	1	4 hours
London	FY2 doctors in secondary care	Teaching hospital	Mandated	30	2 hours
East of England	General practitioners and practice nurses	Primary care	Voluntary	60	30 min
North East England	Multiprofessionals from 1 hospital	Hospital	Voluntary	5	3 hours

*BMJ*, *British Medical Journal*; FY2, foundation year 2; NEPHELP, Nutrition Education Policy in Healthcare Practice.

At the end of the workshop, five (12.5%) NEPHELP evaluation questionnaires were returned from two doctors working in internal medicine, one GP, one nurse and one service manager (table 2). While all respondents agreed that the workshop was useful for themselves and their colleagues, there were suggestions that the workshop included greater involvement of community care and increased focus on elements of nutrition care across the care continuum. Following this feedback, the focus for NEPHELP to also consider general practitioners and other healthcare professionals was widened.

The total response rate for the NEPHELP evaluation tool was 12.5% for the pilot and 10% for subsequent workshops. In addition, 15% completed PGME evaluation tools, and 26% of participants contributed some feedback but the heterogenic nature meant there was limited consistency in evaluation methods between each session. The combined PGME and NEPHELP evaluation data are reported individually in table 3.

**Table 3:** Combined PGME and NEPHELP evaluation data

Total participants	Pilot Subsequent workshops	40 169	Pilot total NEPHELP Q: 5 (12.5%) Total NEPHELP Q: 17 (10%), 1 time interview Total PGME evaluation: 26 (15%) Feedback was received from 26% (excluding the pilot)

NEPHELP, Nutrition Education Policy in Healthcare Practice; PGME, Postgraduate Medical Education.

### The roadshow

Of the six workshops held during the Roadshow, three were mandated sessions and three were voluntary, as summarised in table 2. Mandated sessions had much higher attendance; however, there was a low response rate overall to the evaluation questionnaires. Feedback from each session was used to inform future sessions (table 4).

**Table 4:** Summary of evaluation findings from each workshop

Workshop	Data collection technique	Responses, n, (%)	Key points from the evaluation	Key quotes from the evaluation	Learning points and reflections to inform the next session
*BMJ Health and Safety* conference (pilot; 4 hours)	NEPHELP tool+observations	5 (12.5%)	Nutrition was not adequately addressed in clinical practice (n=2) Identified barriers to nutrition care in practice, including lack of knowledge, time, and a clear scope of practice (clear roles) (n=4) Nutrition education should be a bigger focus of medical training (n=4) Emphasis placed on nutrition in medical education should increase over time (n=3).	Stop talking about discharges, it is a transfer or care, not a discharge. With a change of language hospitals might get with the programme	Workshop needs greater acknowledgement of the role of community care. Need to increase focus on the care continuum, that is, the continuation of nutrition care from hospital to primary or community care. The cases and interactive learning activities had better engagement so more time should be spent on this in future sessions. To increase cases, time for didactic lectures should be decreased.
South East England (2 hours)	PGME evaluation (rating + ‘what was good’ ‘what was not so good’ and ‘suggested improvements’)	8 (24.2%)	Participants scored the session 3.2/4. Positive comments included finding the session interesting, useful, informative and clinically relevant. Some participants appreciated the speaker’s expert knowledge, enthusiasm and likeability.	Informative and clinically relevant” “Good cases and discussion Really good to hear about the new catering contract as this is something relevant to our day-to-day practice and directly affects our patients. The speaker on this topic was particularly engaging. Could have cut out a lot of the history about nutrition and put more focus on what we can do as clinical.	As one participant particularly enjoyed learning from a local dietitian, efforts were made in future sessions to include local expertise. There was a preference for a shorter and more practical session. Content needed to reflect local context, including issues and difficulties faced.
	NEPHELP#	0 (0.0%)			
South East England (2 hours)	PGME evaluation (‘what went well?’ and ‘what could have been improved?’) Ad observations	18 (45.0%)	All 18 respondents gave positive reflections within open text feedback. 6/18 requested more time for nutrition education despite not specifically being asked about this.	Very informative+professional. Difficult confusing topic. Great top tip. Should be in every VTS/school More time! Would love to delve more - esp. Gut talk	Based on previous feedback, the teaching team consisted of one NEPHELP team dietitian in collaboration with a local GP. Positive feedback to have doctor delivering content as role model to others. Need for practical tips/solutions to common issues.
	NEPHELP	0 (0.0%)			
Essex (1 day)	Discussion	1 (100%)	The GP who attended did not perceive that nutrition education would be of interest to colleagues and instead suggested they be referred to dietitians. Time was mentioned as a barrier to the provision of nutrition care, suggesting the role of the GP might be to refer patients to credible nutrition information and websites.		Based on feedback from the previous session, a much longer, yet voluntary session was offered. Unfortunately, the recruitment for this session was very low. Sessions not mandated were less well attended. Need to ensure clinical relevance to attending professionals.
London (2 hours)	NEPHELP and observations	17 (52%)	The workshop somewhat (n=10) or definitely (n=6) helped to develop skills/knowledge to increase their confidence in providing nutritional care. The nutrition education programme would be somewhat (n=10) or definitely (n=5) interesting to their colleagues (n=2 unsure). The most useful element of the training were the case studies (n=11, 65%), followed by content related to nutrition screening and risks (n=7, 41%) and the background information (n=2, 12%). n=9 felt the programme could be improved by focusing on practical and applied case-based exercises and skills such as communication and nutrition assessment.	Don't need so much background. More clinically relevant stuff TPN and NG feeding More tips on conversations to have with patients	The teaching team was broader with three NEPHELP members a local dietitian and junior doctor. Facilitators reflected that more time is needed to discuss participants roles and their perceived barriers to nutrition care. Content to be clinically relevant. Further discussion is needed around strategies to help address barriers and empower the participants in their role. Specific time allocation to complete paper copies of the NEPHELP evaluation during the session rather than via an electronic link improved engagement.
East of England (30 min)	NEPHELP And observations	(0.0%)	No formal evaluation.		This rapid session was delivered as a part of an afternoon primary care staff training session specific to nutrition. Delivered by one NEPHELP educator. The session highlighted key points on nutrition adequacy and conditions related to all forms of malnutrition.
North East England (3 hours)	NEPHELP And observations	(0.0%)	No response.		This session was advertised via hospital internal communications and open to any National Health Service staff in one hospital trust along with a local dietitian and medical doctor. Discussions within the workshop indicated that the content was well received but that attendance had been difficult due to being scheduled during routine working hours, though it was an optional lunchtime session.

GP, General Practitioner; NEPHELP, Nutrition Education Policy in Healthcare Practice; PGME, Postgraduate Medical Education.

Multiple methods for delivery were tested, spanning rapid sessions in existing training, to full day workshops, with no format perceived as being ideal. The variation in format addressed the logistical constraints on time and location, and the need to fit the workshops within existing programmes of study. For example, a full day session requested by a GP trainer (Essex) was held on a weekend, and although advertised and free, only one person attended. In contrast, the rapid session (East of England) attended by 60 participants aimed to see if the training could be added into an existing event, and if all learning objectives could be covered in the shorter time. Formal feedback from this session was limited.

### NEPHELP evaluation responses

The mandated teaching in London at a teaching hospital for foundation year 2 had the highest response rate to the NEPHELP evaluation tool at 52% (n=17). Nine participants (53%) reported receiving no previous nutrition education, while eight (47%) recalled some nutrition training in their medical education. Of participants who reported receiving nutrition training, five reported receiving this education during medical school, one from a self-selected module, one during a biomedical sciences degree and one elsewhere in their foundation training. All participants felt that the clinical nutritional needs of patients were not prioritised, and as a result were poorly addressed in the hospital setting. As noted by one participant, nutrition is ‘*certainly, something that could be improved*’, but felt that it was ‘*unlikely to be top priority in a consultation*’.

When asked to give an example of where nutritional needs for a patient were met, 41% (n=7) recalled examples of acute nutrition, namely, either nasogastric or parenteral nutrition, 29% (n=5) reported seeing clinical benefit from malnutrition treatments and three specifically mentioned the benefits of dietitian involvement in secondary care. One participant noted examples “In upper GI (gastrointestinal) surgery a lot of patients were on TPN (total parenteral nutrition) or NG/NJ (naso gastric/naso jejunal) feeds.” Another mentioned “Gastro ward with dietitian input (eg, TPN patient).”

When these participants were asked about the barriers to effective nutritional care, 15 (88%) identified time as a barrier, 11 (61%) identified a lack of knowledge, 8 (47%) identified a lack of clarity on roles and responsibilities and 7 (41%) perceived a lack of interest from colleagues as a barrier to such care in practice. One participant said, “I'm aware as a junior in A&E [Accident and Emergency] it is quite slow spending time taking a good diet history and giving advice would make consultations even longer.” When questioned about including nutritional screening or history taking as part of their practice respondents reported the following reasons a lack of nutrition training (n=6, 35%), time pressures for doctors (n=5, 29%) and some participants perceived nutrition care as a dietitian’s role (n=4, 24%). Comments included, “Poor training and perception that nutrition is the preserve of dietitians. Time not allocated”. Another focused on the lack of education: “Little education in med school. Not much time. Unclear whose role it is.” The lack of interest and importance was also mentioned: “Both knowledge on importance but also lack of interest in implementing what little people do know about nutrition.”

Most respondents (88%) felt doctors had a role in nutrition care with half (n=9) identifying the role of a doctor in initial assessment to identify nutrition risks and a third (n=5) indicating the importance of onward referral to the dietitian as part of their role. As one participant mentioned, there are “Very few specialists so it needs to be taken responsibility for by all staff.” They saw their role as the “Assessing, advising, signposting and referring.” Another participant focused on actions to take, particularly regarding handover. “Reorganising when and where referrals are required. Risk assessment. Improving inter-team handover.” While one questioned the role of a doctor in nutrition care, highlighting bigger societal issues. “Some roles, but the most significant barriers lie in public health policy, food poverty and education.”

Most participants in this group (16/17) felt that nutrition education should be an essential element of medical training and found the content of the workshop relevant. When asked about the most appropriate timing of this nutrition education, 12 participants (71%) felt this should be linear, with nutrition education increasing from medical school to junior doctor, while 3 participants (18%) felt nutrition education was most relevant to junior doctors. Two participants (12%) felt nutrition training was most relevant to medical students and would be best placed in undergraduate medical education. Seven of the participants (41%) valued skill-based nutrition education and case-based learning. However, opinions varied on what was relevant nutrition content, but included, ‘practical examples and how to put into practice’*, ‘*important from Global public health perspective’, ‘common things in hospital for example, refeeding’, ‘eating disorders, intuitive eating, diet culture and how best to educate without promoting diet culture’. In contrast, one participant did not perceive nutrition as relevant in medical training as “Patient education and school age education plus socioeconomic factors far outweigh medical input” and others felt that “didactic teaching [was] less useful.”

### Mandated versus voluntary attendance

Mandated sessions were better attended and evaluated. In the UK, all junior doctors have protected learning time to attend mandated teaching.^11 13 20 22^ NEPHELP workshops were delivered in mandated teaching during two FY2 training sessions and one GP trainee protected teaching session. In contrast, attendance at non-mandated training was particularly poor. Participants commented on the difficulties experienced in attending non-mandated sessions due to professional and personal pressures. “If I don’t have to go it’s not something I would attend, at the end of a week I have other priorities”.

Training was generally better received when local doctors were present and less well received when other healthcare professionals individually delivered the same content. The perception was that training would have been better received if a medical role model acted as a peer educator, for example, where teaching was codelivered with another GP trainee as opposed to being delivered by non-medical members of the NEPHELP team who are unknown to the participants. Limited opportunity for interprofessional education (IPE) and poor collaboration in nutrition care across health systems was also identified as a barrier to effective nutrition care practice.

### Low prioritisation of nutrition in clinical care

Many participants identified a low priority given to nutrition in clinical care. Foundation year trainees recognised that nutrition is not addressed adequately in clinical practice but did not have recommendations for how it could be improved, citing logistical challenges such as a lack of priority, time and senior recognition of nutrition in patient management, care planning and treatment. They also felt that there was limited time available for education and variation in opinion on the most important aspects of nutrition. Some topics rated as important were based on prior clinical experiences such as working in a ‘gastro’ environment, or seeing TPN/NG feeding, while others perceived a topic to be importance based on personal interests, such as the ‘low carbohydrate diet*’*. Many participants indicated that their personal nutrition knowledge, level of interest and social media informed their practice.

### Lack of clarity on roles and responsibilities in nutrition care

While participants recognised the importance of nutrition, they remained unclear on their role and scope of practice (as doctors) in nutrition care. Participants attending the primary care training day did not feel that a session on nutrition was appropriate for them as GPs and perceived nutrition as relating solely to healthy eating. Many Foundation doctors found it challenging to address nutritional issues in practice and indicated that their focus was the immediate medical concern. Some participants believed that public health professionals should hold more responsibility than doctors in the delivery of nutrition care. Addressing misconceptions about the role of diet in the prevention and management of non-communicable diseases, its role in medical treatment across all settings, and how different members of the multi professional healthcare team can work synergistically to achieve favourable patient outcomes, may raise the profile of nutrition in medical practice.

It is recognised that collaboration across professions such as registered nurses, doctors and allied health professionals fosters a positive and rewarding practice environment and improves patient outcomes.^23^ This collaboration has previously been identified by medical students and junior doctors with an interest in nutrition as a key factor to support the integration of nutrition into medical practice.^24^

Interprofessional education (IPE) is recognised by the WHO as an ‘innovative strategy that will play an important role in mitigating the global health workforce crisis’ and address professional silo working.^25^ The cross-cutting nature of nutrition, and its involvement in the promotion of health and prevention and management of disease, reiterates that nutrition should be a core element of all healthcare professional’s education. Ideally, it should be ideally through an IPE lens to foster a multidisciplinary approach in practice.

### Challenges for multiprofessional learning, working and collaboration

During NEPHELP, there was a reluctance from PGME providers to include non-medical professionals within junior doctor-protected sessions, limiting the opportunity and scope for IPE. Moreover, there was a perception that the NEPHELP workshop was not as well received when a medical doctor was not part of the teaching team. For trainees, the historic paucity of nutrition education within medical training limits the number of role models who can model and advocate for nutrition care.^8 26^ However, research has identified the importance of professional role models to support learning in medical education and how this authenticates the place for content and its relevance to clinical practice.^27^

## Discussion

Using action research methodology, the NEPHELP team set out to develop an educational package based on GMC outcomes for graduates, which could be delivered in different healthcare settings. In addition, they sought to understand where doctors and health professionals see nutrition as fitting into their educational journey and considered the feasibility of this workshop approach.

### Lessons learnt from NEPHELP

From this work, we recognise that there is no one ‘place’ for nutrition, but there is a need for clear curriculum content at all stages of a doctor’s education.

### Undergraduate and postgraduate nutrition curriculum development

The recently published undergraduate nutrition curriculum for medical doctors represents a consensus among multiple stakeholders, nutrition professionals and medical royal colleges on the required nutrition competencies for medical graduate.^28^ This benchmarks what should be taught in medical education as an accompaniment to GMC learning outcomes for graduates. In addition, the 2021 Foundation curriculum^13^ supports engagement with third-sector organisations, which are at the forefront in providing services to support health prevention including services to support education or access to healthy foods or services directly addressing food poverty.

Clearer postgraduate mandated nutrition competencies within existing PGME curriculum may help to better elucidate key nutrition knowledge and skills for practicing doctors, which could be a useful accompaniment to the foundation doctor’s curriculum, helping to increase validity and visibility of nutrition while also clarifying the role of medical doctors and the wider MDT. However, F2 participants completing the NEPHELP evaluation tool indicated a preference for more linear nutrition education, suggesting a desire to advance nutrition along with other medical skills but with a clear preference for more clinically focused, case-based teaching.

To demonstrate interprofessional roles and responsibilities of care, there is a need for clinically relevant scenarios more closely aligned to existing roles and workplace expectations to ‘nudge’ professionals to raise the profile of nutrition in their practice, as part of an MDT approach. Educating doctors in the absence of the MDT may not address the issue.

Organisations such as NNEdPro, the AfN and GMC can work with educators, including medical schools and foundation programmes to create a framework for trainees to work towards achieving the role and competencies they outline.

Furthermore, recent findings from Lepre *et al*,^29^ reflecting the expressed needs of end users within the medical/healthcare workforce, indicate the need for knowledge and skills to consider the findings from nutrition screening and assessment and coordinate nutrition care, thereby highlighting the importance of the findings from this work in implementation.

### Summary of recommendations

#### Nutrition educators

- Nutrition content needs to reflect the context of the workplace, with most participants indicating their preference to more clinically focused practical teaching directly relevant to their roles and signposting to resources the participants can use.
- Participants preferred practical-based/short-based/skills-based education, which can be easily linked back to practice and can be easily integrated into short windows of opportunity for education.
- Universal nutrition education, such as NEPHELP, may offer a short, focused baseline form on which other nutrition education can be recommended to support core as well as more specialist and potentially a more expert specialist nutrition education pathway.

#### Clinicians in primary and secondary care

- The needs of those in primary care and secondary care were noted to be significantly different, as well as a major need to reframe the communication and transfer of nutrition care between the two settings.
- There is a need for IPE or discussion across professional boundaries to support nutrition pathways. Without this, it may be difficult to address some of the identified barriers. This may pose a challenge in finding a common language so that nutrition messaging is clear and breaks through professional silos within a more multiprofessional model of care.

### Postgraduate medical educators

- Although nutrition is mentioned in published curriculum frameworks, examples of how nutrition might be included in education for UK Junior doctors are limited^30^ and should be developed to support capacity building.
- Trainee GPs wanted more in-depth nutrition education suggesting we need a variety of options and opportunities; from the minimum standards to assure patient safety, to potential career pathways for further specialisation.
- To support sustainability, a PGME nutrition curriculum may assist in reaching a consensus on expectations related to nutrition and professional working roles.
- PGME providers can take advantage of existing multidisciplinary nutrition educators such as the UK Nutrition Implementation Coalition. Training workshops also need to be endorsed by a professional body with relevant continued professional education credits.

### Strengths and limitations

Data from multiple sources of feedback were pooled to provide deeper insights into some of the potential barriers and enablers to implementing nutrition education.

Heterogenic feedback forms and processes in each setting limited analysis. Attempts to standardise data collection on participant opinions on the utility of the nutrition workshops, as well as perceptions on nutrition roles and responsibilities in practice, were made. The NEPHELP evaluation tool was not a validated tool, which limited the usefulness of the feedback captured. To minimise facilitator pre conceptions, postworkshop discussions, alongside evaluations from participants, were used.

Another limitation was the recruitment of participants to participate in the non-mandated sessions. This was particularly evident during the Essex Roadshow event, where despite the workshop being organised at the request of the primary care providers and the enthusiasm to run this workshop outside of normal working hours at the weekend, only one participant attended. Generally, due to the nature of nutrition education and its broad application across multiple areas of practice, most health practitioners would be interested in focused nutrition content that relates to their clinical setting, specialty and region. Hence, we would imagine that if the nutrition content is clinically and regionally specific, this might stimulate greater interest and enthusiasm in nutrition education and its application in their healthcare setting.

Demographics of participants completing feedback were mainly junior doctors and general practitioners. The low response rate limits the generalisability and transferability of our findings and does not consider the views of other healthcare professionals regarding enablers and barriers to nutrition education, as well as the provision of nutrition focused care on clinical practice.

## Conclusion

The NEPHELP project successfully delivered and adapted a bespoke nutrition education programme for doctors. However, there was no clear a ‘place’ for this training and there are significant, ongoing, barriers to delivering nutrition training in the medical postgraduate setting.

For this reason, in the UK, NNEdPro, ERimNN (Education And Research In Medical Nutrition Network), Culinary Medicine UK and Nutritank have come together to form the Nutrition Implementation Coalition (2022–23).^31^ A coalition encompassing multiprofessionals who can provide a central hub of material and expertise that will support medical schools and health professionals who may lack the current faculty to develop and implement nutrition education. Also, the multiprofessional nature of the coalition can act as a role model for interprofessional working to focus on developing the seamless delivery of nutritional care. Such coalitions may be one way of developing and sustaining interest in developing nutrition expertise, by work alongside mandatory training across the range of healthcare professionals in both primary and secondary care. Finding opportunities for the delivery of clinically relevant nutrition education in ‘bitesize’ sessions may tap into the need for solution-focused education. Nutrition is central to health and disease in both prevention and treatment, this has been recently highlighted through COVID-19 and its sequelae. The interest that developed during the pandemic could provide an opportunity to translate research back into fundamental nutrition training.

Recognising there is no one ‘place’ for nutrition, the Nutrition Implementation Coalition provides a strategy to provide a ‘hub’ of material and expertise that allows the content to be available yet adapted to the needs of the providers and settings. Beyond this paper, this research has continued with the development of the online NEPHELP course via a virtual learning environment and its evaluation in primary care.

## Data Availability

Data are available upon reasonable request.
